# Adhesive bond strength of monolithic zirconia ceramic finished with various surface treatments

**DOI:** 10.1186/s12903-023-03630-7

**Published:** 2023-11-13

**Authors:** Işıl Sarıkaya, Yeliz Hayran

**Affiliations:** 1 Department of Prosthodontics, Tokat Gaziosmanpaşa University Faculty of Dentistry, Tokat, Turkey; 2 Department of Prosthodontics, Bursa Uludağ University Faculty of Dentistry, Bursa, Turkey

**Keywords:** Monolithic zirconia, Incoris TZI, Adhesive resin cement, Shear bond strength, Thermal aging, Surface roughness

## Abstract

**Background:**

This study aimed to investigate different surface treatments thought to increase the bond strength between zirconia ceramic and adhesive resin cement.

**Methods:**

The samples were prepared in 15 × 10 × 2 mm dimensions by cutting off monolithic zirconia ceramic blocks (Incoris TZI; Sirona, Germany). Surface roughness measurements were made with a profilometer, the average surface roughness (Ra1) was recorded, and five different surface treatments were applied. Group 1: Control group. No surface treatment was applied. Group 2: Sandblasted with Al_2_O_3_ under pressure of 50 μm. Group 3: Sandblasted with 30 μm Al_2_O_3_ - SiOx under pressure, then tribochemical silica coating, silane bonding agent, and ceramic primer were applied. Group 4: Samples were etched in a hot acid solution containing methanol, HCl, and chloride at 100 °C. Group 5: Samples were coated in a solution containing Grade C Aluminum Nitrite at 75 °C for 15 Sects. 12,000 thermal aging was carried out to all samples. Then, samples were bonded to a composite surface (Filtek Z250) with two different types of adhesive cement (Panavia F 2.0, Rely X U200) (n = 10). A load was applied to the samples attached to the Universal Test Device for the SBS, and the SBS was recorded. The surface roughness measurements of all samples were made again, and the average surface roughness Ra2 was recorded. The data was analyzed with a two-way ANOVA test. Bonferroni correction was used for multiple comparisons of the groups. *p* = 0.005 was accepted as the statistically significant value.

**Results:**

There was no statistically significant difference between the groups in the Ra1 measurements (*p* = 0.031). There was a statistically significant difference between the Ra2 values of Groups 4 and 5 and the Ra2 values of Groups 1,2 and 3 in the Ra2 measurements (*p* < 0.001). There was no statistically significant difference between the SBS values of the groups (*p* > 0.005). Also, there was no statistically significant difference in the SBS values of all groups for the two different cements tested (*p* > 0.005).

**Conclusions:**

None of the surface treatments applied to monolithic zirconia ceramic samples increased the SBS between ceramic and adhesive resin cement.

## Background

Zirconia is indicated in restorations where esthetics and resistance are needed together. Monolithic zirconia is becoming a preferred material resistant to occlusal loads, even at a 0.5 mm occlusal distance [1, 2]. However, more than a good connection between the resin cement and zirconium oxide is needed [3].

Suggested surface treatments for zirconium and resin connection are sandblasting, tribochemical silica coating, hydrofluoric acid (HF) etching, and erbium-yttrium aluminum garnet (Er-YAG) laser irritation in the literature [4–6]. Mechanical abrasion techniques are beneficial for achieving increased bond strength on zirconia surfaces [7]. The most commonly used of these surface treatments is sandblasting [8]. This method abounds the zirconium surface with 50 to 250 μm aluminum oxide (Al_2_0_3_) particles. However, this procedure may cause phase changes by creating microcracks on the zirconium surface [9]. It has been reported that resin bonding agents heal small surface cracks caused by sandblasting and strengthen ceramics [10].

Some manufacturers recommend tribochemical silica coating as an alternative process to improve the bond between zirconium oxide (ZrO2) ceramic and resin cement [11]. In the tribochemical silica coating method, the ceramic surface is coated with aluminum oxide modified with silicic acid. During coating, this sand can be embedded in the ceramic surface to a depth of 15 μm. In this way, a glass phase layer is formed on the surface of the ceramic coated with silica. This layer establishes a chemical bond with the silane applied to it [6]. Micromechanical locking and chemical adhesion to ceramic surfaces are essential for a strong resin bond [12]. In the cementation of silica-based ceramics, good bonding can be obtained using hydrofluoric acid (HF) - followed by silanization [10]. In contrast, zirconium is silica-free ceramic resistant to conventional etching techniques [13, 14]. Regarding zirconium oxide and resin cement bonding, HF acid etching does not give satisfactory results because of the high crystal content and glass phase [15]. On the contrary, Altan et al. [16], reported that HF acid etching successfully created a better adhesion to zirconium oxide ceramic surface. To increase and improve the roughened zirconium surface area, laser application [17], selective infiltration technique (SIE) [18, 19], hot acid solution [20], and nanostructured aluminum applications such as nitrite coating (AIN) [21] are investigated in the literature. However, there are various surface treatment methods and studies evaluating the effects of these methods. There is still no consensus regarding the best surface treatment method for optimal bond strength between zirconia ceramic and adhesive resin cement [4].

Primers containing resin cement and 10-methacryloxydadecyl dihydrogen phosphate (MDP) monomer are recommended for adhesive cementation of zirconium restorations. Thus, it reveals a chemical process between the hydroxyl groups of the zirconia ceramic and the phosphate ester monomer between the MDP-containing agents [15, 22, 23].

The thermal aging method is a method that has been used to determine the intraoral behavior of restorative materials under in vitro conditions. The aim is to examine the response of materials to temperature changes in the case of hot and cold applications. The number of thermal cycles, the waiting time in the water, and the pause time vary in research. Generally, thermal aging is applied in conjunction with mechanical loading in analysis. Therefore, the number of thermal cycles depends on the duration of the mechanical test and the dwell and pause time determined by the thermal aging unit [24]. The number of thermal cycles applied in the studies to age the materials varies between 1–1.000.000. In addition, it is accepted that the application between 500 − 10,000 cycles is meaningful [25]. The shear bond test (SBS), widely used to evaluate bond strength, is a simple and reliable in-vitro test [26–28]. Shear force is applied at a speed of 0.75 ± 0.3 mm/min until separation occurs in the connection between the samples consisting of two different materials. Shear resistance per unit area is obtained by dividing the maximum applied force by the joint surface area [29].

Studies examining the bond strength of monolithic zirconia ceramics with adhesive resins are limited in the literature. The study evaluated the adhesive bond strength of zirconia ceramics, in which different surface treatments were applied to resin cement, using thermal cycling and shear bond strength tests. The null hypothesis of this study was that other surface treatments would not improve the SBS of adhesive cement and the monolithic zirconia compared to the control group.

## Methods

Materials used in the study are listed in Table 1.

**Table 1:** Materials used in the study

Materials	Manufacturer	Batch number
**Incoris TZI 40/19**; Monolithic zirconia blocks	Sirona Dental Systems, Bensheim, Germany	20,181,655,596
**Panavia F 2.0**; Adhesive resin cement	Kuraray Medical, Tokyo, Japan	#488-EU 000061
**Rely X Unicem**; Adhesive resin cement	3 M Espe, Seefeld, Germany	3,931,691
**Filtek Z 250;** Universal composite resin	3 M Espe, Minn., USA	33-04855-000- 33-04866-000
**Monobond-N;** Silan bonding agent	Ivoclar Vivadent, Liechtenstein	W85815
**Clearfill;** Ceramic primer	Kuraray, Japan	K010101

### Preparation of samples

In the study, five different surface treatments and two different types of cement were tested on 100 samples (n = 10). Samples were obtained by slicing pre-sintered monolithic zirconia blocks (Incoris TZI; Sirona, Bensheim, Germany) of size 55 / 19 dimensions in a water-cooled precision cutting machine (Micracut 201, Bursa, Turkey) in 2 × 10 × 15 mm dimensions. Sample sizes were prepared 20% larger and adjusted to be 6 × 8 × 12 mm after sintering. All samples were sintered according to the manufacturer’s instructions and air-dried after cleaning with ethanol in an ultrasonic bath (Pro-Sonic 600; Sultan, NJ, USA). Then all samples were ground with 600, 800, 1000, and 1200 grit silicon carbide abrasive papers (3 M Espe; St. Paul, USA) with a water-cooled grinding machine (Metkon Gripo 2 V, Bursa, Turkey), respectively, to obtain standard sample surface-samples stored in distilled water for 15 min in the ultrasonic bath.

### Surface roughness measurements

Surface roughness measurements of the samples were made with a profilometer (Taylor Hobson, Surtronic 25, Leicester, UK). Measurements were made at an evaluation length of 1.25 mm and an evaluation range of 100 μm. Three measurements were taken from each sample, and the average was taken. A constant measurement speed of 0.5 mm/s was used to determine the average roughness profile (Ra1) in µm. Afterward, the samples were stored in distilled water at 50 °C for 10 min in a 28 kHz frequency ultrasonic cleaner (Pro-Sonic 600, Sultan Healthcare, Hackensack, USA). Subsequently, samples were randomly selected and divided into five groups. One hundred samples were prepared, with ten samples in each group (n = 10) (Table 2).

**Table 2:** Sample groups and surface treatments in the study

Groups	Surface treatments	Adhesive Resin Cement	
		Panavia F 2.0	Rely X Unicem
**Group 1**	Control- No treatment	n = 10	n = 10
**Group 2**	Sandblasting	n = 10	n = 10
**Group 3**	Silica coating + Silan + Primer	n = 10	n = 10
**Group 4**	Hot chemical etching	n = 10	n = 10
**Group 5**	AIN coating	n = 10	n = 10

### Surface treatments

#### Group 1

Control group. No surface treatment has been applied.

#### Group 2

Sandblasted with 50 μm Al_2_O_3_ (Korox; Bego, Bremen, Germany) for 15 s at 2.5 bars pressure perpendicular from 10 mm distance to the sample surface.

#### Group 3

Tribochemical silica coating with 30 μm Al_2_O_3_-SiOx (Cojet; 3 M Espe, Seefeld, Germany) for 20 s at 2.5 bars pressure, then applied silane bonding agent (Monobond-N; Ivoclar Vivadent, Liechtenstein), and ceramic primer agent (Clearfil Ceramic Primer; Kuraray, Okayama, Japan).

#### Group 4

Samples were etched in a hot acid solution containing 800 ml methanol, 200 ml HCl, and 2 gr iron chloride at 100 °C for 10 min.

#### Group 5

Samples were coated for 15 s at 75 °C in 250 ml solution containing 3%, 1.2 μm Grade C Aluminum Nitrite (AIN).

### Thermal aging

A total of 12,000 cycles of thermal aging was performed on all samples in the Thermal Cycling Device (Thermocycler, SD Mechatronic, Munich, Germany) to mimic the intraoral conditions. Bath temperatures were set to be five ℃ – 55 ℃, and the waiting time at each temperature was 60 s. The waiting time in the air between the two temperatures was set as 10 s [30].

### Adhesive cementation

Universal composite resin blocks (Filtek Z250, 3 M Espe, Minn, USA) 2 mm -thick were prepared in rectangular prisms by the zirconia ceramic dimensions by the same researcher. One of the types of cement used in this study was the diphosphate monomer and MDP-containing self-etch, dual-cure resin cement Panavia F2.0 (Kuraray Noritake Dental Inc., Japan). By the manufacturer’s recommendation, the alloy primer was first applied to the samples, waited for 30 seconds, and then air-dried. Panavia F Paste A and Paste B were mixed at a ratio of 1:1 with the special spatula included in the set for 20 seconds. The mixed resin cement was placed on the sample surface, and light (Demi Led Light Curing System, Kerr, USA) was applied for 20 seconds after removing the residual cement. Finally, Oxyquard II was used and waited for 3 minutes. Following the manufacturers ' recommendations, the other samples were cemented with Rely X Unicem, a conventional self-etch, dual-cure resin cement containing methacrylates and phosphoric ester groups. During cementation, samples were mounted in a clamp, and a force of 50 N was applied for 5 minutes to mimic finger pressure. Adhesive resin cement types, compositions, and contents are listed in Table 3.

**Table 3:** Adhesive resin cement type, composition and content

Adhesive Resin Cement	Composition	Content
Rely X Unicem	MDP contains resin cement,	Filler 78%, MDP dimethacrylats, initiator
Panavia F 2.0	Conventional resin cement	Filler 72%, dimethacrylates, mathacrylated phosphoric ester

### Shear bond strength test

The measurement of the SBS of the samples was carried out using the Universal Test Device (AGS-X, Shimadzu, Tokyo, Japan). A unique mold was prepared to apply the shear test to the samples, which were kept in distilled water at 37 °C for 24 h after cementation. The tip of the test apparatus, which will perform the cutting process, was adjusted to make an angle of 90 ° with the zirconia ceramic surface in the samples. Then, a shear force was applied to the interface at a 0.5 mm/min speed. The force value at the point where the separation occurred was recorded in Newtons. Newton (N) values were converted to Megapascal (MPa) values to determine the amount of charge per unit area (N / r^2^). Then, surface roughness measurements of all samples were made with the same method as the first measurements, and the average surface roughness (Ra2) was recorded.

### SEM

A random sample was selected from each group. Samples washed with distilled water were dehydrated in a 100% alcohol solution for 30 minutes. After the prepared samples were vacuumed with a vacuum device (Quorum SC7620, Quorum Technologies Ltd, England) in an airless environment, the sample surfaces were coated with Au-Pd. Images were taken under x 500 magnification. This procedure was performed with the SEM device (Jeol JSM-7001F, Japan). Types of fracture; ‘adhesive fracture’ in which the adhesive cement is wholly separated from the zirconia ceramic; ‘cohesive fracture’ in which the adhesive cement completely breaks within itself; and ‘mixed fracture’ (adhesive + cohesive) in which both fracture types are observed.

### Statistical analysis

The sample size calculation was performed using G*Power v. 3.1.9.3 software (Heinrich-Heine-Universitat Dusseldorf, Germany). A sample size, at the level of *α* = 0.05, with an effect size of 0.6 and power of 0.8 was used. Accordingly, a total of at least 100 samples, ten in each group (n = 10), should be studied. Statistical analysis of the study was performed with SPSS 20.0 (SPSS v20.0; IBM SPSS Inc., Chicago, IL, USA). They assessed that all the obtained results were normally distributed, and the differences in the measures in terms of groups were evaluated using repeated measures of Variance analysis. The results are expressed as mean ± standard deviation, and the significance level is 5% (*p* < 0.05).

## Results

A two-way analysis of variance was used for repeated surface roughness measurements. The averages, standard deviations (SD), and differences between groups for the mean surface roughness values (Ra1, Ra2) of all samples’ measurement results are shown in Table 4. A statistically significant difference existed between groups in the Ra1 measurements (*p* = 0.031). There was a statistically significant difference between the Ra2 values of Groups 4 and 5 and the Ra2 values of Groups’ 1,2, and 3 (*p* < 0.001). There was no statistically significant difference in intra-groups in the Ra1 and Ra2 measurements of Group 1 (*p* = 0.194). However, there was a statistically significant difference in intra-groups in the Ra1 and Ra2 measurements of Groups 2, 3, 4, and 5 (*p* < 0.001).

**Table 4:** Mean values, standard deviations (SD), and intergoups comparisons for Surface Roughness (Ra) of the specimens

	Group 1 (Control)	Group 2	Group 3	Group 4	Group 5	*p*_*1*_
**Ra 1**	0,19 ± 0,11 (a)	0,15 ± 0,05 (ab)	0,14 ± 0,05 (ab)	0,13 ± 0,06 (b)	0,15 ± 0,06 (ab)	0,031
**Ra 2**	0,15 ± 0,1 (a)	0,39 ± 0,18 (c)	0,26 ± 0,08 (b)	0,23 ± 0,1 (ab)	0,25 ± 0,1 (ab)	< 0,001
***p***_***2***_	0,194	< 0,001	< 0,001	< 0,001	0,002	

**p1**: The statistical significance value of the between-groups comparisons,

***p2***: *The statistical significance value of the intra-groups comparisons,*

***(abc)***: *A common letter as a line indicates statistical insignificance*

The averages, standard deviations (SD), and differences between groups for the mean SBS of all samples’ measurement results are shown in Table 5. Bonferroni correction was used for the multiple comparisons of the groups and listed in Table 6. The highest SBS value was recorded in the Group 4 (18.06 ± 8.08 MPa) samples cemented with Rely X Unicem. The lowest SBS value was recorded in the Group 5 (8.79 ± 2.56 MPa) samples cemented with Rely X Unicem. There was no statistically significant difference between all groups in the SBS measurements (*p* > 0.05).

**Table 5:** Mean values, standard deviations (SD), and intergoups comparisons for Shear Bond Strength values (MPa) of the specimens

	Rely X	Panavia
**Group 1**	11,2 ± 0,98 a,x	11,19 ± 1,21 a,x
**Group 2**	11,28 ± 1,24 a,x	13,74 ± 1,84 a,x
**Group 3**	10,43 ± 4,41 a,x	9,33 ± 1,64 a,xy
**Group 4**	18,06 ± 8,08 ab,x	16,11 ± 2,05 a,x
**Group 5**	8,79 ± 2,56 a,x	10,34 ± 5,17 a,x

**(ab)**: A common letter as a coloumn indicates statistical insignificance

***(xy)***: *A common letter as a line indicates statistical insignificance*

**Table 6:** Multiple comparison of Shear Bond Strength values (MPa) of groups

	(I) Material	(J) Material	Average Difference (I-J)	SS	*p* ^**^	95% Confidence Interval of the Difference	
						Lower limit	Upper limit
**Rely X**	Group 1	Group 2	-,080	2,969	1,000	-9,444	9,283
		Group 3	,776		1,000	-8,588	10,139
		Group 4	-6,857		,317	-16,221	2,506
		Group 5	2,419		1,000	-6,945	11,782
	Group 2	Group 1	-,080		1,000	-9,444	9,283
		Group 3	,856		1,000	-8,507	10,220
		Group 4	-6,777		,336	-16,140	2,587
		Group 5	2,499		1,000	-6,865	11,862
	Group 3	Group 1	-,776		1,000	-10,139	8,588
		Group 2	-,856		1,000	-10,220	8,507
		Group 4	-7,633		,182	-16,996	1,731
		Group 5	1,643		1,000	-7,721	11,006
	Group 4	Group 1	6,857		,317	-2,506	16,221
		Group 2	6,777		,336	-2,587	16,140
		Group 3	7,633		,182	-1,731	16,996
		Group 5	9,276		,053	-,088	18,639
	Group 5	Group 1	-2,419		1,000	-11,782	6,945
		Group 2	-2,499		1,000	-11,862	6,865
		Group 3	-1,643		1,000	-11,006	7,721
		Group 4	-9,276		,053	-18,639	,088
	Group 1	Group 2	-2,553	2,969	1,000	-11,916	6,811
		Group 3	1,856		1,000	-7,507	11,220
		Group 4	-4,925		1,000	-14,289	4,438
		Group 5	,852		1,000	-8,512	10,215
	Group 2	Group 1	2,553		1,000	-6,811	11,916
		Group 3	4,409		1,000	-4,954	13,773
		Group 4	-2,373		1,000	-11,736	6,991
		Group 5	3,404		1,000	-5,959	12,768
	Group 3	Group 1	-1,856		1,000	-11,220	7,507
**Panavia**		Group 2	-4,409		1,000	-13,773	4,954
		Group 4	-6,782		,334	-16,145	2,582
		Group 5	-1,005		1,000	-10,368	8,359
	Group 4	Group 1	4,925		1,000	-4,438	14,289
		Group 2	2,373		1,000	-6,991	11,736
		Group 3	6,782		,334	-2,582	16,145
		Group 5	5,777		,659	-3,587	15,140
	Group 5	Group 1	-,852		1,000	-10,215	8,512
		Group 2	-3,404		1,000	-12,768	5,959
		Group 3	1,005		1,000	-8,359	10,368
		Group 4	-5,777		,659	-15,140	3,587

* 0.05 level of significance ** Bonferroni correction for multiple comparisons

### Fracture type analysis findings by SEM

**Fig. 1:**
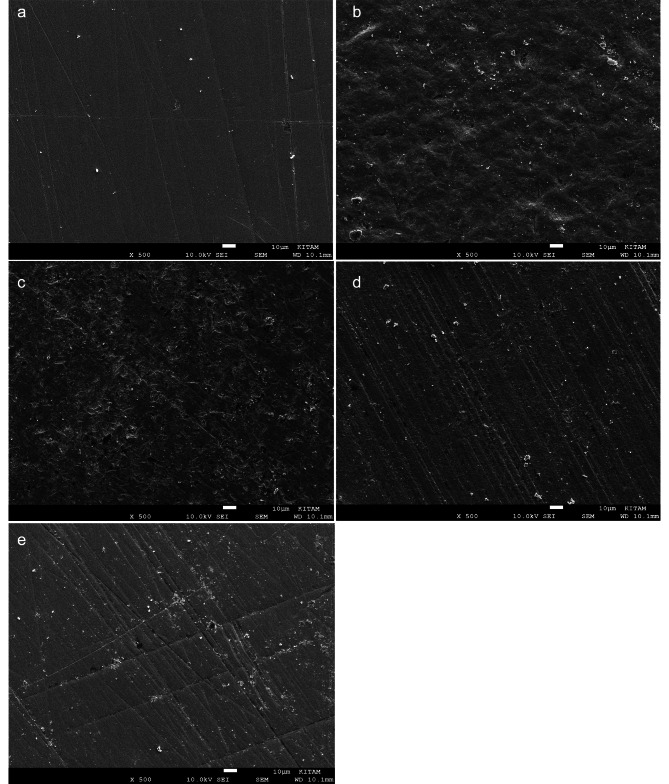
SEM images of x 500 magnifications of the monolithic zirconia ceramic surfaces’ finished with various surface treatments: **(a)** Control (G1) **(b)** Sandblasting (G2) **(c)** Silica coating + silane + ceramic primer (G3) **(d)** Etching in hot acidic solution (G4) **e**: Aluminum nitrite coating (G5) Note the surface lines are similar with a, c, d, and (e) Note that microporosites on the ceramic surface are seen in b, c, d, and e. No resin cement or composite residue is observed on the surfaces with adhesive failure

Representative SEM images of x 500 magnifications of the monolithic zirconia ceramic surfaces’ finished with various surface treatments are shown in Fig. 1. Types of fracture for all samples were observed as ‘adhesive fracture’ in which the adhesive cement is wholly separated from the zirconia ceramic.

## Discussion

The study aimed to compare different surface treatments that are thought to increase the bond strength of zirconia ceramic and adhesive resin cement. The null hypothesis of this study was that other surface treatments would not improve the SBS of the resin cement and the monolithic zirconia, compared with the control group with no surface treatment applied. There was no statistically significant difference in the SBS values of all groups for the two adhesive resin cement tested. Therefore, the hypothesis was accepted in light of the present data.

In the present study, Ra1 measurements were similar in each group, but Ra2 measurements were found to be different for intragroups and intergroups except for the control groups. All four surface treatments used in the study were influential in creating roughness bonding surfaces. Up to the results of the present study’s surface roughness measurements, AIN-coated Incoris TZI surfaces have presented similar roughened surfaces with Incoris TZI specimens, etched in a hot acidic solution.

The lowest and the highest SBS values were obtained in the Rely X Unicem cemented groups in zirconia ceramics. SBS values of all groups were found to have statistically similar results in MPa. In the present study, SBS values of groups in MPa were higher than 10 MPa except for two groups, as a common denominator even after thermocycling [27]. Physiochemical conditioning methods, such as air-abrasion protocols, abrasives or etchants, and silane or primer, have been accepted to increase the bond strength of resin cement to zirconia [27]. Aging with thermal cycling can be considered an effective technique for handling reliable results for in vitro testing of MDP-containing resin cement bonding to zirconia ceramics, in line with the study results. Studies on SBS of zirconia ceramics in the literature have reported that lower SBS values in MPa were obtained in those aged by thermal cycle [27, 28]. Özcan et al. [31], reported that the adhesion of Panavia F2.0 on zirconia decreased after 6000 thermal cycles. A total of 12,000 thermal cycles were used in the present study. This cycle’s number may affect the bond strength of the cemented specimens of Panavia F2.0. The number of cycles may explain all fractures seen as the adhesive fracture type in the present study during thermal aging.

SBS is a macro test method that reflects the clinical situation [27, 28]. Microtensile bond strength (MTBS) is a more sensitive test. The SBS test method, faced with micro-tensile bond testing, can produce non-uniform stresses [32]. These erratic stresses can cause cohesive failure, leading to inaccurate results [33]. It requires fine sectioning of a material for testing micro tensile bonding. Taking thin sections without breaking is challenging when sintered Zr and resin cement are bonded. Also, Valandro et al. [34], reported that both tests give parallel results for alumina-zirconia ceramics (In-Ceram Zirconia). They concluded that both chairside and laboratory types of tribochemical silica coating followed by silanization showed higher bond strength results when compared to aluminum oxide abrasion and silanization, regardless both of the test methods-MTBS test, and SBS, test employed.

Akay et al. [35], reported that the hot chemical etching technique had successfully strengthened the shear bonding of conventional (Variolink II) and MDP-containing adhesive resin cement (Panavia SA) on zirconia ceramics. Both of the adhesive resin cements used in this study contain MDP. According to the study results, there was no superiority over each other in obtaining a solid bond strength between both types of cement. MDP-containing bonding/silane coupling agents can increase bonding on zirconia ceramic surfaces because MDP monomer can diffuse to hydroxyl groups of ceramics, and chemical bonding can occur in this way. Atsu et al. [15], investigated SBS of zirconium oxide ceramic and composite resin cemented with Panavia F adhesive resin cement (does not contain MDP). They have reported that the highest bond strength of 22.9 ± 3.1 MPa was obtained with the silica coating, and the combination of bonding/silane coupling agent groups was applied. The specimens’ highest bond strength in the present study was 18,06 ± 8,08 MPa, etched in hot acidic solution (G4) cemented with Rely X Unicem cement containing MDP. Although higher MPa values have been handled with Atsu et al.’s [15] study, comparing the present study, thermal aging has not been applied in their study, even ignoring the material difference investigated.

Jevnikar et al. [21], reported that the AIN coating technique effectively increased SBS between resin cement and Y-TZP ceramics. In their study, similar to the present study, MDP contained- Rely X Unicem was used, and 12,000 thermal cycling was applied to the specimens. This study detected that Rely X Unicem’s bonding capacity for adhesive cementation with zirconium was at the highest level but not meaningful. SEM images obtained from the samples in our study suggest that the AIN coating may be similar to sandblasting, tribochemical silane coating, and etching with a hot acidic solution. This finding is in agreement with previous results [27, 28]. Also, due to the different surface treatments in the present study, SEM images didn’t show remarkable surface irregularity or any micro retentive grooves.

Behr et al. [36], investigated the SBS and tensile bond strength (TBS) of the zirconia-to-resin bonding after sandblasting the surfaces by applying 12,000 thermal cycling. The surface treatments used a silane coupling agent, tribological silica coating (Rocatec system), types of cement or primers containing different types, and the proportion of phosphates. They have reported that silane coupling agents alone showed shallow TBS values. Silica coating was only sufficient when combined with phosphate esters and postpone-containing primers. Based on diphosphates (Panavia F 2.0) or postpones (Multilink sprint), bonding agents recorded TBS values under 10 MPa. They have considered that bond strength higher than 10 MPa was clinically sufficient. Also, they concluded that none of the investigated bonding concepts of the zirconia-to-resin interface provided clinically sufficient TBS. SBS values were inadequate for a sufficient ranking. In the present study, all the SBS values found over 10 MPa of the groups of Incoris TZI zirconia ceramics, except for the groups which AIN coated and cemented with Rely X Unicem G5 (8,79 ± 2,56), and Panavia G5 (9,33 ± 1,64).

Sandblasting creates microcracks in the zirconium ceramic’s surface and increases the strength of the ceramic [9]. The silica coating creates a glassy layer on the ceramic surface. This layer forms a chemical bond with the silane [6]. Tribochemical silica coating bonds to silane better than sandblasting, providing chemical retention with its silica-coated Zr surface [16, 34]. The highest bond strength can be obtained by melting a glass matrix and crystal surfaces with the HF acid etching method [5]. Applying HF acid and silane to the surface before cementation provides good bonding for silica-based ceramics [10, 37]. However, HF acid etching is unsuccessful in high-strength alumina and zirconia-strengthened ceramics due to the absence of a glassy phase or high crystalline content [18]. However, Altan et al. [16], reported that the application of HF acid to the Incoris TZI monolithic ceramic surface increased the surface energy and wettability of zirconium and, as a result, increased SBS compared to the control group. However, Ural et al. [38], reported that HF acid application did not cause any change in the surface morphology of zirconia.

When several studies evaluated various surface treatment methods on bond strength between zirconia ceramic and adhesive resin cement, there still needs to be consensus in the literature. Also, a study using the same techniques and materials has yet to be found in the literature. In other trials, the number of thermal cycles may give different results for adhesive cements. One of the limitations of the study was, that only a total of 12, 000 thermal cycles were applied to the specimens. Also, since thin sections could not be taken from the study samples, a micro tensile bonding test could not be applied, which may be another limitation of the study. In future studies, consider comparing the effectiveness of the study results with the micro tensile bonding test.

## Conclusion

The conclusions drawn from our study are as follows:

1. There was no difference in the average surface roughness of all ceramic samples before applying the surface treatment in the “control” and “aluminum nitrite coating” groups. The aluminum nitrite coating technique is ineffective on the Incoris TZI ceramic surface for surface roughness.
2. In terms of surface roughness, sandblasting, silica coating, and etching in hot acidic solution were effective in all samples, but no difference was observed compared to each other.
3. SBS to Incoris TZI monolithic zirconium ceramic was the same for both Panavia and Rely X types of cement.
4. There was no difference in the SBS for both cementations with two different adhesives to Incoris TZI monolithic zirconium ceramic between sandblasting, silica coating, etching in a hot acidic solution, and aluminum nitrite coating technique.

## Data Availability

The datasets used and analyzed during the current study are available from the corresponding author upon reasonable request.

## Abbreviations

**µm**: Micrometer
**Al**_**2**_**O**_**3**_: Aluminum
**SiOx**: Silicon Oxide
**HCl**: hydrochloric acid
**°C**: Centigrade degrees
**sec**: Second
**SBS**: Shear Bond Strength
**Ra**: Surface roughness value
**mm**: Millimeter
**HF**: Hydrofluoric acid
**Er-YAG**: Erbium YAG
**ZrO**_**2**_: Zirconium dioxide
**SIE**: Selective Infiltrative Etching
**AIN**: Aluminum Nitrite
**MDP**: 10-methacryloxydadecyl dihydrogen phosphate
**kHz**: KiloHertz
**ml**: Mililiter
**gr**: Gram
**%**: Percent
**N**: Newton
**MPa**: Megapascal
**r**: Radius
**Au-Pd**: Gold-Palladium
